# BMC Bioinformatics comes of age 

**DOI:** 10.1186/1471-2105-6-140

**Published:** 2005-06-07

**Authors:** Matthew J Cockerill

**Affiliations:** 1 Director of Operations, BioMed Central Ltd, Middlesex House, 34-42 Cleveland Street, London, W1T 4LB, UK

## 

Almost exactly five years ago, in early June 2000, BMC Bioinformatics received its first submission. Five years on, it has received over a thousand submissions, and the journal is continuing to grow rapidly (Figure 1).

In the past few months, developments have included a refreshed international editorial board, which now consists of over 50 leaders in the field, and a Bioinformatics and Genomics gateway that brings together relevant content from across BioMed Central's 130+ Open Access journals. And by the time you read this,  *BMC Bioinformatics* should have its first official ISI Impact Factor. Impact factors certainly have their problems – a previous editorial in this journal[1] discussed the arbitrariness of the process by which ISI selects journals for tracking, and the resulting unnecessary time delay before Impact Factors become available. One thing is clear though – with *BMC Bioinformatics* having an Impact Factor, there are more reasons than ever to make it the first choice for your research.

## Five years in bioinformatics

Looking back over the first 5 years of the journal, are any significant trends evident? One thing that is noticeable is the prevalence of the open-source model of software development. In fact more than 10% of all BMC Bioinformatics articles include the term "open-source". Hundreds of open-source bioinformatics projects are now hosted on sites such as bioinformatics.org and sourceforge.net. No doubt the similar philosophies of open-source software and Open Access publishing have been a factor in making *BMC Bioinformatics* one of BioMed Central's most successful journals. Two other emerging trends are, firstly, an increasing use of web service technology to connect disparate tools into analysis pipelines, and secondly, the development of systems to allow biological knowledge to be modelled and expressed in structured form. The linking factor between both these trends is that increasingly, as the data deluge continues, the 'users' of bioinformatics tools and the 'readers' of the biological literature, are likely to be computer systems rather than human beings. 

## Web services and data analysis pipelines

As bioinformatics tools have proliferated, the complexity of data analysis has increased. Often, a sequence of analysis steps each using different tools must be carried out one after the other. This might be done manually or by using a monolithic system that is capable of carrying out multiple analyses, or, more flexibly, by writing special 'glue code', often in Perl, to connect together multiple tools into a pipeline. The problem with the latter approach, though, is that in the absence of defined standards for the input and output of different tools, lots of glue code has to be written in order to create each new pipeline. Worse, systems built in this way tend  to be fragile, since at any time one of the tools in the pipeline may change the format of its input or output (breaking the system), because there is no explicit 'contract' between the various tools as to what input and output formats each will support. Web services [2], and more generally, 'Service Oriented Architectures' [3] promise to provide a solution by providing a means for codifying standard interfaces that can be used to expose bioinformatics tools over the web. Projects such as MyGrid [4] have then built on these standards to provide biologists with graphical user interfaces that can be used to build new analysis pipelines interactively, without needing to write code. *BMC Bioinformatics* has published several articles on the use of Web Service technologies such as the Simple Object Access Protocol (SOAP) - if you are interested, try searching the journal for: SOAP OR "web services"

## Text mining and biological semantics

Another growth area in bioinformatics has been the structured representation and modelling of biological knowledge. The Gene Ontology project [5] has provided an important foundation for much of this work, defining a set of controlled vocabularies that allow biological concepts and relationships to be expressed in a standard way.

Much of the initial work on modelling biological knowledge has explored the use of text-mining techniques to automatically derive structured semantic information from the relatively unstructured text of scientific research articles. BioMed Central's Open Access corpus[6] is now rapidly approaching 10,000 articles and provides ideal raw material for such research.. It is already being used by many researchers, both in industry and academia.

*BMC Bioinformatics* publishes many  papers on text-mining topics, including the recently published supplement [7], which consists of papers presented at last year's BioCreAtIvE text-mining workshop in Granada, Spain. Text mining has its limits, however. Imagine what could be achieved if articles, rather than consisting entirely of free-form natural language, contained explicit assertions about biological knowledge in unambiguous, machine-readable form. This is the oft-vaunted promise of the ‘Semantic Web’ [8], but it has proved to be very difficult to realize in practice.

Some recent developments, however, suggest that progress is being made. For example, this editorial was created using Publicon[9]- a new breed of scientific authoring tool developed by Wolfram Research with input from BioMed Central. Publicon is easy to use, but it is also a highly structured authoring environment. It can not only output BioMed Central's native article XML format, but also embed mathematical equations as 'islands' of semantically-rich MathML [10]. This structured mathematical information is then preserved throughout the publication process, from the author's computer right through to the reader's desktop with no intermediate unstructured version along the way that might cause information to be lost.

So, for example, if you are accessing this editorial online using a suitable browser, you should be able to cut and paste the equation below into any MathML-aware application, as a mathematically meaningful equation rather than an image.

$$\left(i\;\nabla -m\right)\;\Phi_{e^{2}}[B,x]=B(x)\;\Phi_{e^{2}}[B,x]+i\;e^{2}\;\gamma_{\mu }\;\int \delta_{+}(s_{x\;1}^{2})\;\left({\delta \Phi }_{e^{2}}[B,x]/\delta \;B_{\mu }(1)\right)\;\mathrm{d}\tau_{1}$$

In two accompanying Commentaries, the issues associated with capturing and representing biological knowledge are discussed further. Murray-Rust *et al.*[11] consider how chemical information can best be represented within scientific articles, and what bioinformaticists and chemists can learn from one another. Meanwhile, Mons [12] explores in more detail how smart authoring tools can enrich the scientific literature by allowing authors to express themselves unambiguously, avoiding the 'data burying' that makes text mining necessary in the first place.

## Figures and Tables

**Figure 1:**
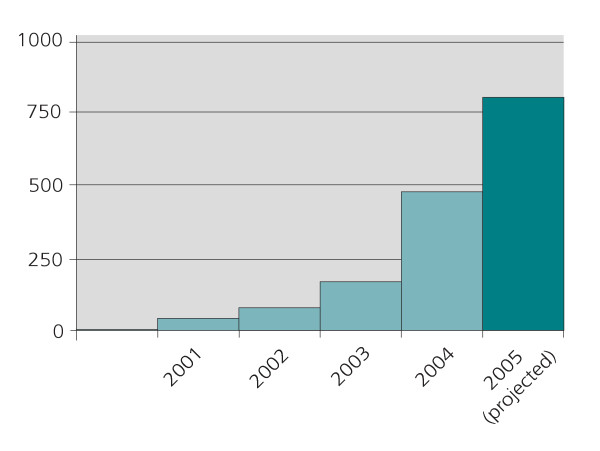
Number of submissions to BMC Bioinformatics. The figure for 2005 represents a conservative projection based on the rate of growth of submissions during the first half of the year.
